# FvWRKY75 Positively Regulates *FvCRK5* to Enhance Salt Stress Tolerance

**DOI:** 10.3390/plants14121804

**Published:** 2025-06-12

**Authors:** Shan Li, Yi Jiang, Hanxiu Xie, Kangwei Wang, Kebang Yang, Qian Cao, Hao Xue

**Affiliations:** 1 School of Horticulture, Anhui Agricultural University, Hefei 230036, China; 19960653711@163.com (S.L.); 18726139679@139.com (Y.J.); 19156213306@163.com (H.X.); wkw19961209@icloud.com (K.W.); 16634254430@163.com (Q.C.); 2 Shanghai Agricultural Technology Extension Service Center, Shanghai 201103, China; 15721351226@163.com

**Keywords:** strawberry, salt stress, FvWRKY75, *FvCRK5*, *Arabidopsis thaliana*

## Abstract

Strawberry (*Fragaria × ananassa* Duch.) is an important fruit worldwide whose growth, development, and productivity are threatened by salinity. The WRKY transcription factors (TFs) were reported to play an important role in regulating abiotic stresses response. However, research on their roles to regulate salt stress tolerance in strawberry remains limited. In current study, the *FvWRKY75* gene was isolated and characterized from the Ruegen strawberry, and induced by various stress treatment. The results showed that the FvWRKY75 transcription factor was a transcriptional activator and localized in the nucleus. Phenotypic and physiological analysis revealed that ectopic expression of *FvWRKY75* in *Arabidopsis* improved salt tolerance by enhancing the antioxidant system activities, modulating ROS scavenging and upregulating stress-related genes. Y1H and dual luciferase assays revealed that FvWRKY75 can directly bind to the promoter of the *FvCRK5* gene by recognizing the W-box element. Compared with the WT, ectopic expression of *FvCRK5* gene in *Arabidopsis* enhanced salt tolerance characterized by the reduced ROS accumulation, higher chlorophyll content, lower MDA content, and enhanced SOD and POD activity. Herein, the FvWRKY75 gene acted as a positive regulator in salt stress resistance, at least in part, via the WRKY-CRK network to regulate the antioxidant enzyme defense system and stress-related genes to regulate salt stress tolerance in strawberry.

## 1. Introduction

Strawberry (*Fragaria × ananassa* Duch.) is an important fruit species for its delicious, rich nutrition and high economic value [1]. China is the largest producer of strawberry in the world, and the majority yield of strawberry is produced in facility cultivation. In strawberry production regions, overfertilization, improper irrigation, and environmental pollution in greenhouses have exacerbated secondary salt damage, which adversely affects the yield and quality of strawberry fruit [2].

Plants require suitable environmental conditions to grow, such as optimum temperature, light, water, and soil nutrients. However, soil salinization and secondary salinization (salt stress) has become one of the major negative factors restricting agricultural production and quality because of the intensification of global warming and desertification [3,4,5]. High salt concentrations may result in a series of physiochemical changes in plants (such as osmotic stress, ion toxicity, secondary oxidative stress, etc.), and even lead to plant mortality due to nutrient imbalance, water deficiency, and energy depletion [6]. Plants usually generate various approaches to defend salt stress, including stomatal closure, reactive oxygen species (ROS) clearance, accumulation of osmotic adjustment substance, ionic balance, hormone synthesis, and response [7,8,9]. The process by which plants respond to salt stress involves a complex regulatory network, and many transcription factors, including WRKY, NAC, bZIP, BHLH, and MYB families, have been proven to play essential roles in resistance to salt stress [10,11,12,13].

Many reports have proved that the WRKY transcription factors family is one of the largest transcription factor families in higher plants, which can participate in numerous biological pathways during plant growth and development stage [14,15,16,17]. Additionally, many reports have shown that the WRKY transcription factors play a critical role in the regulation of the abiotic stress response [18,19]. Overexpression of *TaWRKY33* and *LiWRKY39* in *Arabidopsis* increases the thermotolerance by improving the expression level of stress-related genes [20,21]. The knockout *OsWRKY76* gene in rice dramatically decreases cold tolerance by repressing the expression of cold-induced genes [22]. Overexpression of *PtrWRKY75* in poplar and *SpWRKY8* in tomato enhances drought tolerance by regulating stomatal conductance and improving water-use efficiency [23,24]. Overexpression of *DgWRKY3* in tobacco, *DcWRKY3*, *DcWRKY5*, *DcWRKY12, VvWRKY30, GhWRKY34,* and *FvWRKY42* in *Arabidopsis*, *DgWRKY5* in chrysanthemum, *ZmWRKY104* in maize, and *AhWRKY75* in peanut enhances salt tolerance by improving antioxidant enzyme activity, reducing ROS accumulation, and activating stress-related genes’ expression [25,26,27,28,29,30,31,32,33].

The complex regulatory mechanism of WRKY TFs exposed to biotic and abiotic stresses in strawberry is still poorly understood. To date, 62 *FvWRKY* genes and 222 *FaWRKY* genes have been identified in the diploid wild strawberry and octaploid cultivated strawberry, while some *WRKY* genes have been reported to play a different role in biotic and abiotic stress responses [34,35,36,37,38]. However, these previous reports have not been deeply studied. Therefore, the regulatory role of *FvWRKY*s underlying salt stress tolerance in strawberry needs to be further explored. In the current study, we cloned the *FvWRKY75* gene from the diploid strawberry Ruegen, and found that the *FvWRKY75* gene was significantly induced by salt stress. The resistance to salt stress was increased in *FvWRKY75*-overexpression plants by activation of the antioxidant defense system and stress-related genes. Further analysis revealed that FvWRKY75 directly bind to the W-box element of the *FvCRK5* promoter region. Overall, the results of this study may help to reveal the molecular mechanism of WRKYs in strawberry’s resistance exposure to salt stress.

## 2. Results

### 2.1. Isolation of FvWRKY75 and Sequence Analysis

The CDS sequence of the *FvWRKY75* gene was isolated from leaves of the diploid woodland strawberry Ruegen according to the RNA-seq and Genome Database for Rosaceae (https://www.rosaceae.org/) method. The CDS of the *FvWRKY75* gene was 573 bp, encoding 190 amino acids with an isoelectric point and a molecular weight of 9.45 and 21.67 kDa, respectively.

**Figure 1:**
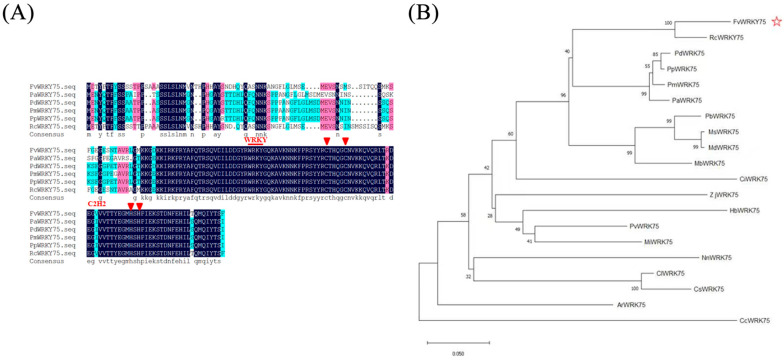
Analysis of the FvWRKY75 transcription factor. (**A**) Amino acid sequence alignment of FvWRKY75; (**B**) phylogenetic tree analysis of FvWRKY75.

The amino acid sequence analysis showed that the sequence of FvWRKY75 presented a highly conserved WRKY DNA-binding domain, and the zinc-finger pattern was the C_2_H_2_ motif (Figure 1A). The phylogenetic tree analysis revealed that the sequence of FvWRKY75 had the highest identity match with RcWRKY75 (*Rosa chinensis*) (Figure 1B).

### 2.2. Subcellular Localization and Transcriptional Activity of FvWRKY75

**Figure 2:**
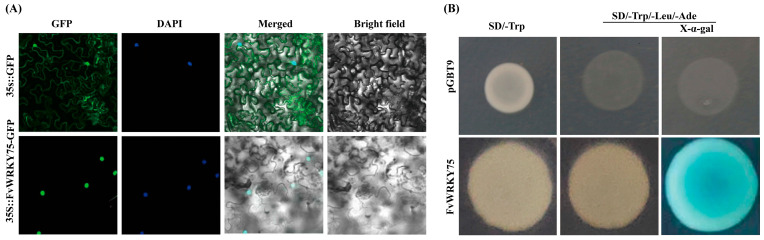
Analysis of the subcellular localization and transcriptional activity of FvWRKY75 transcription factor. (**A**) Subcellular localization of FvWRKY75 in tobacco leaves; (**B**) transcriptional activity analysis of FvWRKY75.

To determine the subcellular location and transcriptional activation of the FvWRKY75 transcription factor, the fusion plasmids for 35S:: FvWRKY75-GFP and BD-FvWRKY75 were constructed. As shown in Figure 2A, the fluorescence of the FvWRKY75-GFP fusion protein was presented only in the nucleus, but the fluorescence of the GFP empty vector was spread throughout the whole cell. Yeast cells transformed with the BD-FvWRKY75 fusion plasmid survived on the SD/-Leu/-Trp/-His medium and changed to blue when the selection medium was supplemented with 20 mM X-a-Gal, while the pGBT9 empty vector failed to grow on SD/-Leu/-Trp/-His medium (Figure 2B). All of these results indicated that the FvWRKY75 transcription factor was a transcriptional activator and localized in the nucleus.

### 2.3. The Expression Profiles of FvWRKY75 Gene

**Figure 3:**
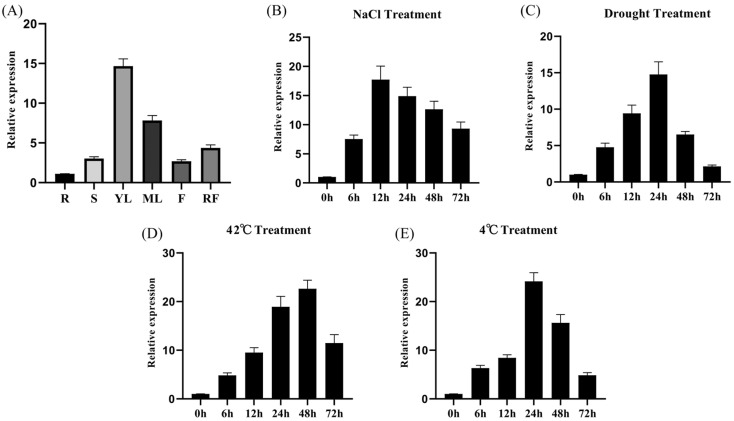
The expression profile of *FvWRKY75* in strawberry. (**A**) Analysis of the tissue-specific expression patterns of *FvWRKY75* in Ruegen strawberry. (**B**–**E**) Expression patterns of *FvWRKY75* in Ruegen strawberry leaves treated with 200 mM NaCl, 20% PEG-6000, and 42 °C and 4 °C treatment. Three independent biological replicates were performed. Data are the means ± SD.

The expression patterns of the *FvWRKY75* gene in different Ruegen strawberry organs and various stress treatments were examined by qRT-PCR to explore their potential functions. As shown in Figure 3, the abundance of *FvWRKY75* transcripts was high in the young leaves and mature leaves; the expression levels of the *FvWRKY75* gene were significantly induced by drought, salt, heat, and cold treatment.

### 2.4. Overexpression of FvWRKY75 Improves the Salt Tolerance in Transgenic Plants

The FvWRKY75-overexpressing transgenic *Arabidopsis* was generated by Agrobacterium-mediated transformation. Thus, two positive transgenic lines were obtained and identified by PCR and qRT-PCR (Supplementary Figure S1). In addition, the *Atwrky75* mutant plants and complementary *FvWRKY75* (CP) lines were also acquired for further testing (Supplementary Figure S1).

**Figure 4:**
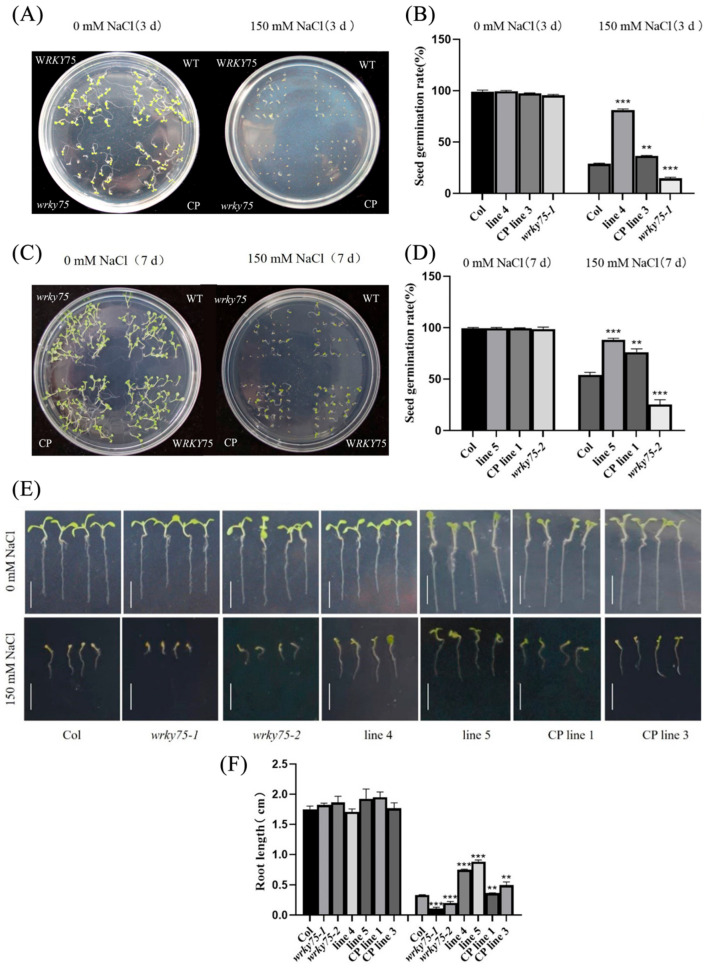
Overexpression of *FvWRKY75* improves the salt tolerance in transgenic *Arabidopsis* seedlings. (**A**–**D**) The germination rate of WT, *Atwrky75* mutant, CP and OE *Arabidopsis* seeds with salt treatment for 3 d and 7 d; (**E**,**F**) root length in each *Arabidopsis* seedlings under salt treatment for 7 d. Three independent biological replicates were performed. Data are the means ± SD. ** *p* < 0.01, *** *p* < 0.001.

**Figure 5:**
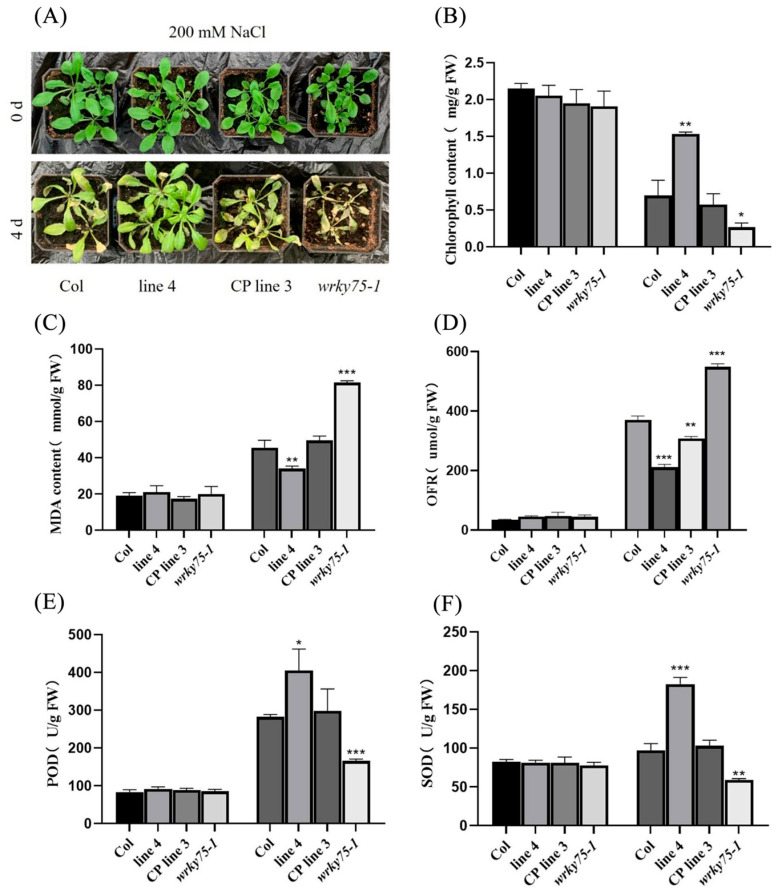
Overexpression of *FvWRKY75* improves the salt tolerance in transgenic *Arabidopsis* mature plants. (**A**) Phenotypes of WT, mutant, CP and OE mature plants with salt stress treatment; (**B**–**F**) chlorophyll content, MDA content, O^2−^ content, SOD activity, and POD activity in mature plants under salt stress, respectively. Three independent biological replicates were performed. Data are the means ± SD. * *p* < 0.05, ** *p* < 0.01, *** *p* < 0.001.

WT, *Atwrky75* mutant, *FvWRKY75* transgenic plants, and the CP lines mentioned above were selected for salt tolerance analyses. Interestingly, the survival rate of the transgenic plants seed was significantly higher than that of the other three types of lines under salt stress treatment (150 mM NaCl), especially that of the mutant seeds with the lowest level (Figure 4A–D). Meanwhile, the root lengths of *FvWRKY75* transgenic plants were markedly different from those of the WT and mutant plants (Figure 4E,F). Thirty-day-old WT, mutant, transgenic plants and CP lines with consistent growth were treated with 200 mM NaCl for 4 d. In particular, the WT and *AtWRKY75* mutant plants showed greater sensitivity to salt stress, while transgenic plants exhibited better performance (Figure 5A). The *FvWRKY75* transgenic plants had lower MDA and O^2−^ contents but higher chlorophyll content and SOD and POD activities compared to the other three types (Figure 5B–F). All of the above showed that the overexpression of the *FvWRKY75* gene can improve salt tolerance in transgenic *Arabidopsis*.

### 2.5. Overexpression of FvWRKY75 Upregulates the Stress-Related Genes

**Figure 6:**
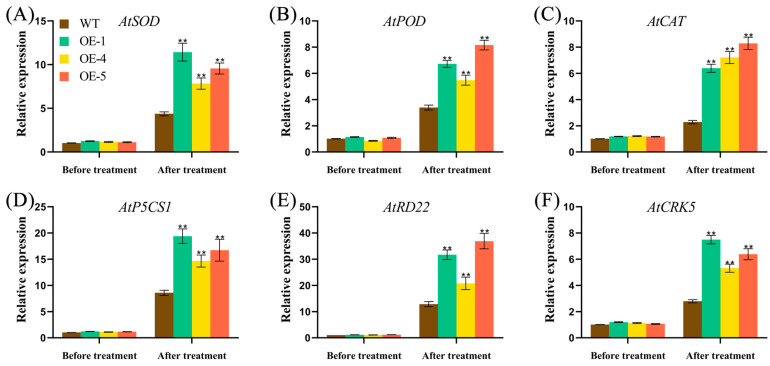
Overexpression of *FvWRKY75* upregulates the stress-related genes under salt stress. qRT-PCR analysis was used to test the expression of various stress-related genes. (**A**–**F**) The transcript level of *AtPOD*, *AtSOD*, *AtCAT*, *AtP5CS1*, *AtRD22*, and *AtCRK5* gene in mature plants under salt stress, respectively. Three independent biological replicates were performed. Data are the means ± SD. ** *p* < 0.01.

Stress-related genes (*AtPOD*, *AtSOD*, *AtCAT*, *AtP5CS1*, *AtDREB2A,* and *AtCRK5*) were selected and we detected the expression levels in WT and *FvWRKY75-*OE transgenic lines by qRT-PCR analysis (Figure 6). Compared with WT, the expression level of those stress-related genes increased significantly in *FvWRKY75*-OE lines under salt stress treatment. Meanwhile, the expression of those genes in *FvWRKY75* OE lines remained at higher levels compared to those in WT seedlings (*p* < 0.01).

### 2.6. FvWRKY75 Can Activate the Promoter of FvCRK5 Gene

**Figure 7:**
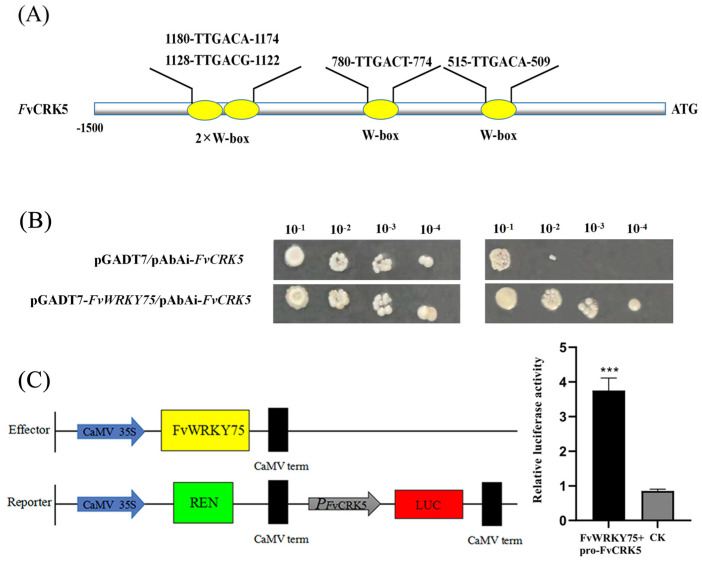
FvWRKY75 activates the expression of *FvCRK5*. (**A**) Bioinformatics analysis showed that the promoter of *FvCRK5* contained WRKY binding elements. **(B)** Analysis of the activation of *FvCRK5* transcription by FvWRKY75 in a yeast one-hybrid assay. (**C**) Luciferase activity assay showed that FvWRKY75 binds to the *FvCRK5* promoter. Data are the means ± SD. *** *p* < 0.001.

In this study, *FvSOD3*, *FvPOD17,* and *FvCRK5,* whose promoter contains the W-box element (TGAC), were selected to determine the regulation relationship with FvWRKY75. As shown in Figure 7B, the FvWRKY75 protein could only directly bind to the FvCRK5 promoter by yeast one-hybrid assay. The effector-reporter assay indicated that the relative luciferase activity of tobacco leaves, which was cotransformed with FvWRKY75 and pro*-FvCRK5,* was significantly stronger than that of the control (Figure 7C). Therefore, the results suggested that FvWRKY75 can activate the promoter of *FvCRK5* to influence its expression.

### 2.7. Overexpression of FvCRK5 Increases the Salt Tolerance in Transgenic Plants

To explore the function of *FvCRK5* gene, 2 FvCRK5-overexpressing transgenic *Arabidopsis* lines were obtained and used for subsequent experiments. The *FvCRK5* transgenic plants were identified by PCR and qRT-PCR (Supplementary Figure S2).

**Figure 8:**
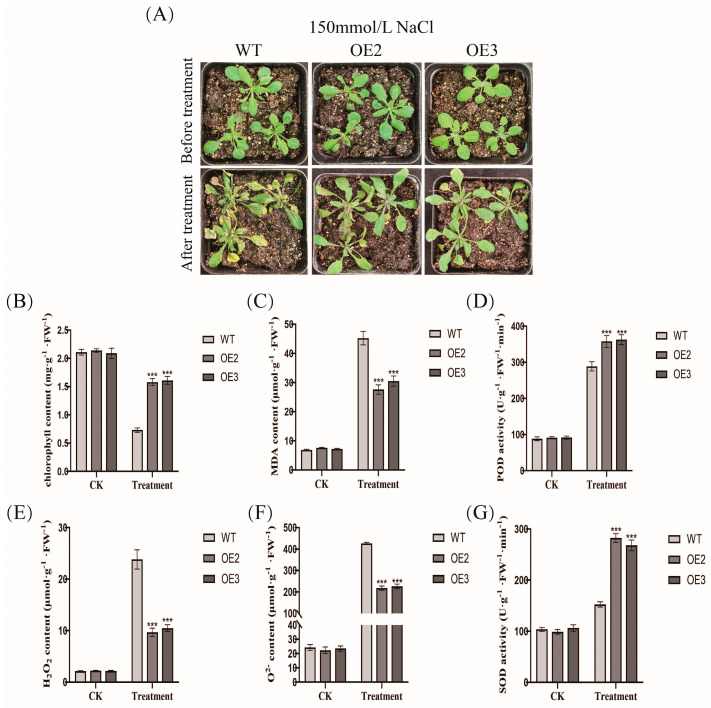
Overexpression of *FvCRK5* increases the salt tolerance in transgenic *Arabidopsis* mature plants. (**A**) Phenotype of WT and *FvCRK5* transgenic mature plants with salt stress treatment; (**B**–**G**) chlorophyll content, MDA content, POD activity, H_2_O_2_ content, O^2−^ content, and SOD activity in mature plants under salt stress, respectively. Data are the means ± SD. *** *p* < 0.001.

After salt stress treatment, *FvCRK5* transgenic plants presented better phenotypes; the transgenic plants had lower leaf yellowing rates than WT (Figure 8A). Inconsistent with the phenotype results, the leaves of *FvCRK5* transgenic plants had higher chlorophyll content, accumulated lower ROS levels and MDA content, and had enhanced SOD and POD activities compared to WT plants, which revealed that minor damage was induced in transgenic plants by salt stress treatment (Figure 8B–G). Thus, those results showed that the overexpression of *FvCRK5* can increase the tolerance of *Arabidopsis* to salt stress.

## 3. Discussion

Higher plants have evolved multiple mechanisms to adapt to changing environmental conditions. In plants, transcriptional factors (TFs) such as WRKY, NAC, bZIP, BHLH, MYB, etc., often generally act as molecular switches in response to stress conditions at the earliest stress signal transduction stage, and control the expression of several downstream genes by binding to the specific elements in their promoters [9,11].

According to previous studies, many WRKY transcription factors have been identified to play important roles in the regulation of various biotic and abiotic stress responses. For example, overexpression of *AtWRKY75* in *Arabidopsis* and *MdWRKY75e* in apple increases resistance to *Pectobacterium carotovora* ssp. *Carotovora (Pcc) and Alternaria alternata* [39,40]; overexpression of *PtrWRKY75* in poplar and *AhWRKY75* in peanut improves drought and salt tolerance [23,41]. However, the function of the *FvWRKY75* gene’s exposure to salt stress in strawberry is still poorly understood. In the present study, FvWRKY75 contained the complete WRKY DNA-binding domain, and acted as a transcriptional activator localized in the nucleus (Figure 1 and Figure 2), which is consistent with the features of WRKY transcription factors [18]. The *FvWRKY75* gene was expressed in all strawberry tissues and was significantly upregulated under salt, drought, heat, and cold treatment (Figure 3). Those results indicated that *FvWRKY75* may have involvement in the abiotic stress response in strawberry.

Our study demonstrated that the *FvWRKY75* gene is involved in the salt stress response. Under salt stress treatment, the seed germination rate and root length of *FvWRKY75*-OE lines were significantly higher than that of WT, CP, and *Atwrky75* mutant plants (Figure 4). Meanwhile, the performance of *FvWRKY75*-OE lines was better than that of WT and CP lines under salt stress treatment, and mutant plants proved more sensitive to salt stress (Figure 5A). Chlorophyll and MDA content are important indicators for assessing the damage degree of abiotic stress resistance in plants [42]. Under salt stress treatment, higher chlorophyll and lower MDA content was observed in *FvWRKY75*-OE lines, suggesting that *FvWRKY75* attenuates the cellular damage to plants (Figure 5B,C). Those results indicated that the *FvWRKY75* gene acts as a positive regulator of salt resistance.

Under various abiotic stresses, ROS accumulation (particularly H_2_O_2_ and O_2_^−^) in plants causes damage to plant cellular structures and components, which can be scavenged by antioxidant enzymes, including SOD, POD, and CAT [43,44,45,46]. In peanut, *AhWRKY75* transgenic lines exhibit better salt tolerance by improving the efficiency of the ROS scavenging system [41]. Additionally, overexpression of *DgWRKY3* in tobacco, and *DcWRKY3*, *DcWRKY5,* and *DcWRKY12* in *Arabidopsis*, improves salt tolerance through increasing the antioxidant enzyme activity and reducing the reactive oxygen species (ROS) accumulation [25,31,32,33]. In the current study, SOD and POD activity was increased, and the content of O_2_^−^ was decreased in *FvWRKY75*-OE lines, while in *Atwrky75* mutant plants these indicators were exactly the opposite under salt stress, which showed that *FvWRKY75* could promote the removal of ROS by improving the activities of antioxidant enzymes, thus reducing damage to plants caused by salt stress (Figure 5D–F). Furthermore, the expression of various genes encoding antioxidant enzymes and genes involved in ROS scavenging were measured by qRT-PCR. Under salt stress, those stress-related genes were significantly induced in *FvWRKY75*-OE lines compared to WT plants, which suggested that *FvWRKY75* could upregulate the expression of stress-related genes in response salt stress (Figure 6).

Calcium-dependent protein kinases (CDPKs), as essential regulatory proteins of the signal transduction pathway, participate in regulating various abiotic stresses in plants [47,48,49,50,51,52]. In tobacco, overexpression of *AhCDPK* and *GhCDPK4* genes enhances the resistance to salt stress by improving the activity of antioxidant defense systems, and reducing the accumulation of ROS [53,54]. CRK (CDPK-related kinase), a member of the CDPK superfamily, whose C-terminal domain has sequence homology with the regulatory domain of CDPK, cannot bind to Ca [48,55,56,57]. However, the function of the *CRK* gene’s response to salt stress has not been clearly verified. In the current study, FvWRKY75 was directly bound to the promoter of *FvCRK5* by YIH and LUC (Figure 7). Meanwhile, ectopic expression of *FvCRK5* in *Arabidopsis-*enhanced salt tolerance was characterized by the reduced ROS accumulation, higher chlorophyll content, lower MDA content, and enhanced SOD and POD activity compared to that of WT (Figure 8). The *crk1* mutant *Arabidopsis* plants showed increased susceptibility to salt stress under changed MDA content and proline accumulation [58]. Therefore, we speculate that the *FvCRK5* gene, activated by FvWRKY75, plays an active role in the response to salt stress.

In conclusion, we suggest a regulation model of the WRKY-CRK transcriptional regulatory cascade in regulating salt stress in strawberry. In response to salt stress, FvWRKY75 acts as a positive regulator to improve salt tolerance by modulating ROS scavenging, enhancing the antioxidant enzyme activity, regulating stress-related genes’ expression, and directly activating the expression of the *FvCRK5* gene. These results provide physiological and molecular evidence to demonstrate the significance of *FvWRKY75* in plants’ response to salt tolerance.

## 4. Materials and Methods

### 4.1. Plant Materials, Growth Conditions, and Stress Treatment

Ruegen strawberry (*Fragia vesca* L.), tobacco (*Nicotiana tobacum* L.), and *Arabidopsis thaliana* Columbia-0 (Col-0) plants were used in this study, and all seedlings were grown in a growth chamber (23 °C, 4000 lux light, 12 h light/12 h dark, 60% humidity) at Anhui Agricultural University (Hefei, China). Different tissues of Ruegen strawberry seedlings were collected and used to analyze the expression profile of the *FvWRKY75* gene. For heat and cold treatment, two-month-old seedlings of Ruegen strawberry were transferred to 42 °C or 4 °C growth chamber; for salt treatment, strawberry seedlings were irrigated with 200 mM NaCl until saturation; for drought treatment, strawberry seedlings were treated with 20% polyethylene glycol-6000 (PEG-6000, Sigma, Saint Louis, MO, USA). Mature leaves from the above treatments were collected at 0 h, 6 h, 12 h, 24 h, 48 h, and 72 h. All of the leaf samples were frozen in liquid nitrogen and stored at −80 °C.

### 4.2. Structure and Sequence Analysis of FvWRKY75

The full-length sequence of *FvWRKY75* (XM_004310052.2) was downloaded from the Genome Database for Rosaceae (https://www.rosaceae.org, accessed on 10 April 2022) and cloned from Ruegen cDNA using the specific primers (Supplemental Table S1). The amplified fragments were transferred into pMD19-T (TaKaRa, Dalian, China) and sequenced. The DANMAN (version 6.0) software was used to compare the amino acid sequences of FvWRKY75 (accessed on 20 May 2022). The phylogenetic tree was constructed with the MEGA (version 7.0) using the maximum-likelihood (ML) method (accessed on 20 May 2022).

### 4.3. Vector Construction and Genetic Transformation

The full-length CDS region of *FvWRKY75* was amplified and inserted into the pRI101-AN vector with the CaMV 35S promoter, named the pRI101-FvWRKY75 vector. The *FvWRKY75* transgenic lines were obtained according to the methods previously described by the established protocols [59]. The *FvWRKY75* transgenic lines were selected by 30 mg/L kanamycin and confirmed by RT-PCR and qRT-PCR with the specific primers. All of the primers used are listed in Table S1.

### 4.4. Subcellular Localization of FvWRKY75 Protein

The *FvWRKY75* CDS region minus the stop codon was amplified and inserted into the pRI101-eGFP vector to construct the 35S:: FvWRKY75-eGFP fusion vector. The 35S:: FvWRKY75-eGFP fusion plasmid was introduced into tobacco leaves through *A. tumefaciens* GV3101 before observation of the GFP signal by fluorescence confocal microscopy using a Leica TCS SP8 (Leica, Germany) after 48 h darkness. The 35S::eGFP empty vector was used as the control. The methods of the subcellular localization assay used in the current study were previously described in the established protocols [60]. All of the primers used are listed in Table S1.

### 4.5. Transcriptional Activation Analysis

The CDS region of *FvWRKY75* was inserted into the pGBKT7 vector (named BD-FvWRKY75) and transferred to the Y2H strain. The pGBKT7-53 vector served as a positive control. The methods of transcriptional activation assay used in the current study were previously described in the established protocols [60]. All of the primers used are listed in Table S1.

### 4.6. RNA Extraction and Quantitative Real-Time PCR Analysis

Total RNA was isolated from mature leaves or tissues samples by the CTAB methods. The cDNA synthesis and quantitative real-time PCR (qRT-PCR) were performed according to the established protocols [60]. *FvActin* and *Atactin* primers were used as the internal reference for the strawberry and *Arabidopsis* samples, respectively. All of the primers used are listed in Table S1.

### 4.7. Salt Stress Treatment

After surface sterilization, seeds of T3 homozygous transgenic lines, mutant, CP, and WT lines were seeded on 1/2 MS medium with or without 150 mM NaCl. The germination rate and root length were measured with treatments for 7 days. Four-week-old seedlings were watered with or without 150 and 200 mM NaCl for 4 days, then the physiological changes were photographed and measured.

The content of chlorophyll, MDA, H_2_O_2_ and O_2_^−^, as well as the enzyme activity of SOD and POD, was determined by previously reported methods [60].

### 4.8. Yeast One-Hybrid Assay

The CDS region of *FvWRKY75* was cloned and inserted into the pGADT7 vector with the GAL4 activation domain. The promoter of *FvSOD3, FvPOD17,* and *FvCRK5* was cloned and inserted into the pAbAi vector. All transformed yeast cells were cultured at 28 °C for 2–3 days. All of the primers used are listed in Table S1.

### 4.9. Dual Luciferase Activity Assay

The pGreenII-62SK-FvWRKY75 vectors were constructed and defined as an effector. The promoter of *FvCRK5* was inserted into the pGreenII-0800-LUC vector and defined as a reporter. The pGreenII-0800-LUC vector served as a control (Table S1). Dual luciferase activity assays were conducted according to the previously reported methods [60]. All of the primers used are listed in Table S1.

### 4.10. Statistical Analysis

All data are the average of three repeated experiments. SPSS Version 20 was used for data analysis. Values are shown as the means ± SD. After one-way analysis of variance (ANOVA), Duncan’s multiple range test was used to identify significant differences among treatment means (* *p* < 0.05, ** *p* < 0.01 and *** *p* < 0.001).

## Data Availability

Data are contained within the article and can be made available on request.

## Supplementary Materials

The following supporting information can be downloaded at https://www.mdpi.com/article/10.3390/plants14121804/s1, Figure S1: The identification of transgenic *Arabidopsis thaliana* lines.; Table S1: Primer sequences used in this study.
