# Supplementary material for: FvWRKY75 Positively Regulates FvCRK5 to Enhance Salt Stress Tolerance
Source: Plants (Basel). 2025 Jun 12;14(12):1804. doi: 10.3390/plants14121804 (PMC12196777; doi:10.3390/plants14121804)
Supplement: Supplementary file 1 [file plants-14-01804-s001.zip › plants-3627976-supplementary.pdf]

Supplementary materials

Supplementary Figure 1. The identification of transgenic *Arabidopsis thaliana* lines.

Supplementary Table 1. Primer sequences used in this study.

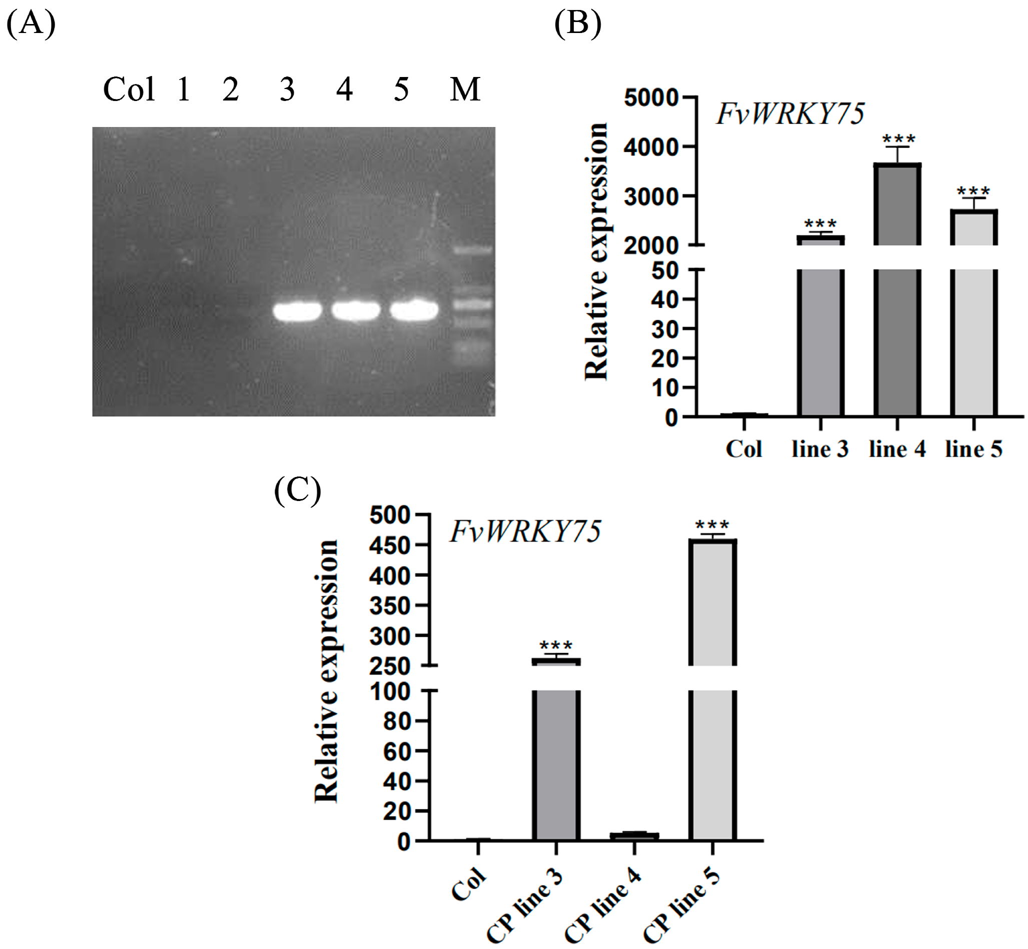

Figure S1. The identification of transgenic *Arabidopsis thaliana* lines. (**A**): PCR assay of *FvWRKY75* transgenic lines; (**B**): Relative expression of *FvWRKY75* gene in different transgenic lines; (**C**): Relative expression of *FvWRKY75* gene in different complementing lines. Data are the means ± SD. *** $p < 0.001$.

Note: Col: Wild-type *Arabidopsis thaliana*; 1: line 1; 2: line 2; 3: line 3; 4: line 4; 5: line 5.

Table S1. Primer sequences used in this study.

Gene name	Primer sequence (5′ to 3′)
FvWRKY75-RT	Forward: ATGGATACCTACCCAACATTCTATT<br>Reverse: TCAGAAAGAAGTGTAGATTTGCATC
FvWRKY75-GFP	Forward: CCATGGATGGATACCTACCCAACATTCTA<br>Reverse: ACTAGTGAAAGAAGTGTAGATTTGCAT
FvWRKY75-BD	Forward: GAATTCATGGATACCTACCCAACATTCTATT<br>Reverse: CTGCAGTCAGAAAGAAGTGTAGATTTGCATC
FvWRKY75-qRT	Forward: CTTCTTCATCAACGCCACCTTCTGC<br>Reverse: ATCTTCTTGTCTCCCTTCTTCATCCCTAA
Fvactin-qRT	Forward: ACC GTT GAT TCG CAC AAT TGG TCA TCG<br>Reverse: TAC TGC GGG TCG GCA ATC GGA CG
Atactin-qRT	Forward: GAAATCACAGCACTTGCACC<br>Reverse: AAGCCTTTGATCTTGAGAGC
AtCAT-qRT	Forward: TGGCAACTACCCCGAGTGGA<br>Reverse: AGAACCAAGCGACCAACCGG
AtPOD-qRT	Forward: GACCCTACACTCAACACTAC<br>Reverse: TTCATTGCCTCCACGAATGC
AtSOD-qRT	Forward: TGGCGAAAGGAGTTGCAGTT<br>Reverse: GGCCAGAAACTGTTCCACTCACA
AtRD22-qRT	Forward: TGGACACAGGCAAGGGCAAG<br>Reverse: CGTCACACCGCCTTTACCGA
AtP5CS1-qRT	Forward: AGGGAAAGTTCCAGAAAG<br>Reverse: CATAACTAAGCGAGCCAC
AtCRK5-qRT	Forward: TTACCTCAAAAGAAGACACCTCT<br>Reverse: ACAATCACACCAACACTCCAAAT
FvNAC114-AD	Forward: GAATTCATGGAGAGCACCGACTCGTCT<br>Reverse: GGATCCCTAAGAATACCAATTCCCCGGA
FvWRKY75-PAbAi	Forward: GAGCTCCGGTGAAGATGTGAAAATTC<br>Reverse: CTCGAGCAAAAAACAGAGAGGTGGAT
pGreenII-SK-FvNAC114	Forward: GAGCTCATGGAGAGCACCGACTCGTCT<br>Reverse:GAATTCCTAAGAATACCAATTCCCCGGA
pGreenII-0800-FvWRKY75	Forward: GTCGACCGGTGAAGATGTGAAAATTC<br>Reverse: GGATCCCAAAAAACAGAGAGGTGGAT
